# Spherical Indentation Behavior of DD6 Single-Crystal Nickel-Based Superalloy via Crystal Plasticity Finite Element Simulation

**DOI:** 10.3390/ma18153662

**Published:** 2025-08-04

**Authors:** Xin Hao, Peng Zhang, Hao Xing, Mengchun You, Erqiang Liu, Xuegang Xing, Gesheng Xiao, Yongxi Tian

**Affiliations:** 1 College of Applied Science, Taiyuan University of Science and Technology, Taiyuan 030024, China; haoxin@tyust.edu.cn (X.H.);; 2 Shanxi Research Center of Basic Discipline of Mechanics, Taiyuan 030024, China; 3 Shanxi Key Laboratory of Material Strength and Structural Impact, College of Aeronautics and Astronautics, Taiyuan University of Technology, Taiyuan 030024, China

**Keywords:** single-crystal nickel-based superalloy, spherical indentation, crystal plasticity simulation, slip behavior, crystal orientation

## Abstract

Nickel-based superalloys are widely utilized in critical hot-end components, such as aeroengine turbine blades, owing to their exceptional high-temperature strength, creep resistance, and oxidation resistance. During service, these components are frequently subjected to complex localized loading, leading to non-uniform plastic deformation and microstructure evolution within the material. Combining nanoindentation experiments with the crystal plasticity finite element method (CPFEM), this study systematically investigates the effects of loading rate and crystal orientation on the elastoplastic deformation of DD6 alloy under spherical indenter loading. The results indicate that the maximum indentation depth increases and hardness decreases with prolonged loading time, exhibiting a significant strain rate strengthening effect. The CPFEM model incorporating dislocation density effectively simulates the nonlinear characteristics of the nanoindentation process and elucidates the evolution of dislocation density and slip system strength with indentation depth. At low loading rates, both dislocation density and slip system strength increase with loading time. Significant differences in mechanical behavior are observed across different crystal orientations, which correspond to the extent of lattice rotation during texture evolution. For the [111] orientation, crystal rotation is concentrated and highly regular, while the [001] orientation shows uniform texture evolution. This demonstrates that anisotropy governs the deformation mechanism through differential slip system activation and texture evolution.

## 1. Introduction

Nickel-based superalloys, as critical materials in modern industry, must simultaneously withstand the synergistic effects of high temperatures, complex loads, and corrosion in extreme service environments such as aerospace power systems [1,2], high-temperature components of nuclear power equipment [3], and corrosion-resistant devices in petrochemical applications [4]. Such environments impose stringent demands on material strength, creep resistance, fatigue resistance, and microstructural stability [5,6]. The strengthening mechanisms of nickel-based alloys primarily arise from the synergistic interactions of γ′-phase ordering strengthening, grain boundary carbide dispersion strengthening, and multiscale dislocation networks [7,8]. The cooperative effects of these microstructural features manifest as superior mechanical properties at the macroscopic level.

Nanoindentation technology, as a critical technique for micro-nano mechanical characterization, provides unique advantages for studying material mechanical behavior at micro/nano scales due to its sub-micron spatial resolution and nN-level load precision [9,10,11]. Guo, D et al. [12] systematically analyzed the indentation creep characteristics of LCZ (The LCZ nanopowder was synthesized by chemical co-precipitation method as raw powder, reconstructed through steps such as ball milling, spray drying, sintering, and plasma treatment, and then prepared by atmospheric plasma spraying (APS).) coatings via nanoindentation, offering foundational support for further research on creep properties of nanostructured LCZ coatings. Ji, XK et al. [13] characterized the influence of loading rate on the power law constitutive parameters of nickel-based single-crystal superalloys (NBSX) using nanoindentation, effectively mitigating interference from indentation sink-in/pile-up effects and indentation size effects. Wang, JP et al. [14] established idealized atomistic models of typical microstructures in nickel-based single crystals, conducting molecular dynamics simulations of nanoindentation. Their work investigated deformation behaviors and mechanisms under localized loading, analyzed dislocation nucleation and propagation, and developed models based on dislocation motion to describe localized plasticity, thereby providing theoretical insights into microstructural evolution. During indentation processes, it remains experimentally challenging to directly observe dynamic evolution phenomena such as three-dimensional stress distribution, dislocation slip band formation, and grain-grain interactions [15]. Furthermore, experimental results are substantially affected by multiple factors, including indenter geometry, strain rate effects, and surface roughness [16,17,18]. These complexities contribute to uncertainties in establishing clear correlations between localized mechanical responses and material microstructural characteristics.

To address these challenges, CPFEM quantitatively describes plastic deformation mechanisms at the microscale and their correlation with material mechanical responses by introducing the critical resolved shear stress criterion for dislocation slip systems and hardening constitutive models [19,20]. Employing CPFEM based on real three-dimensional microstructures, Wang, D et al. [21] systematically investigated texture evolution and slip modes in titanium alloys during cold rolling. Zhao, Q et al. [22] utilized CPFEM to elucidate stress formation mechanisms in monocrystalline and bicrystals at specific oriented grains and grain boundaries within AA2024 alloy. Through studies on the microscopic deformation behavior of GH4169 superalloy, Wu, JG et al. [23] summarized a face-centered cubic (FCC) crystal plasticity constitutive model based on dislocation slip, validating the reliability of CPFE simulations and revealing room-temperature creep mechanisms dominated by dislocation slip. Combining experiments with CPFEM simulations, Han, QN et al. [24] uncovered the influence mechanisms of crystal orientation on pile-up patterns, dominant slip system distribution, and geometrically necessary dislocation (GND) density distribution. Zhou, X et al. [25] employed CPFEM simulations combined with experiments to explain the formation mechanism of gradient polycrystalline copper. Crucially, CPFEM not only reconstructs the nonlinear characteristics of load–displacement curves in nanoindentation experiments but also analyzes key microscopic deformation mechanisms such as lattice rotation induced by crystalline dislocation motion, spatial distribution of slip bands, and evolution of the Schmid factor [26].

Spherical indentation serves as a vital method for investigating the elastoplastic deformation characteristics of materials during indentation. To study the elastoplastic mechanical behavior of nickel-based single-crystal superalloy DD6 (with its detailed chemical composition parameters listed in Table 1) during indentation deformation, nanoindentation experiments combined with CPFEM were employed to systematically investigate mechanical behavior under varying loading rates during spherical indentation. Based on the dimensionless analysis methodology established by Ni, W et al. [27], a multi-parametric coupled energy-stiffness equation incorporating initial unloading stiffness was formulated, enabling analytical solutions for contact area, reduced modulus, and hardness. CPFEM simulations characterized deformation features at different crystallographic orientations and loading rates, while revealing microstructural texture evolution characteristics under varying crystallographic orientations.

## 2. Basic Theory of Crystal Plasticity

Crystal plasticity theory describes the plastic deformation of crystalline materials based on physical mechanisms at the micro/meso scales. This framework is primarily composed of kinematic equations, constitutive relationships, and hardening criteria [28]. At any material point within a grain, the deformation gradient *F* undergoes multiplicative decomposition into the following [29,30]:

(1) $$F=F^{e}\cdot F^{P},$$

where *F***^p^** represents plastic distortion induced by crystalline slip, while *F***^e^** encompasses elastic deformation combined with rigid-body rotation of the crystal lattice. The plastic deformation gradient *F***^p^** satisfies the evolution equation:

(2) $$F^{P}•{\left(F^{P}\right)}^{-1}=\sum_{\alpha }{\dot{\gamma }}^{\alpha }m^{\alpha }⊗n^{\alpha },$$

where ${\dot{\gamma }}^{\alpha }$ denotes the shear rate on slip system, and $m^{\alpha }$ and $n^{\alpha }$ represent the slip direction vector and slip plane normal vector in the plastically deformed configuration, respectively. Assuming that crystal slip obeys Schmid’s law, whereby the slip rate ${\dot{\gamma }}^{\alpha }$ on any slip system depends solely on the projection of stress onto that system [31], the resolved Schmid stress—when neglecting elastic distortion of the crystal lattice—equates to the critical resolved shear stress $\tau^{\left(\alpha \right)}$, defined as follows:

(3) $$\tau^{\left(\alpha \right)}=m^{\left(\alpha \right)}\cdot \sigma \cdot s^{\left(\alpha \right)}.$$

Based on Schmid’s law, the shear rate ${\dot{\gamma }}^{\alpha }$ on slip system *α* is determined by its corresponding critical resolved shear stress $\tau^{\left(\alpha \right)}$ [32]:

(4) $${\dot{\gamma }}^{\left(\alpha \right)}={\dot{\gamma }}_{0}^{\left(\alpha \right)}\mathrm{sgn}\left(\tau^{\left(\alpha \right)}/g^{\left(\alpha \right)}\right){\left|\tau^{\left(\alpha \right)}/g^{\left(\alpha \right)}\right|}^{n}.$$

where $g^{\left(\alpha \right)}$ is the current strength variable of the slip system and *n* is the rate sensitivity factor. Strain hardening is described by the incremental Voce model in terms of strength ${\dot{g}}^{\left(\alpha \right)}$ [33]:

(5) $${\dot{g}}^{\left(\alpha \right)}=\sum_{\beta }h_{\alpha \beta }\left|{\dot{\gamma }}^{\beta }\right|.$$

where $h_{\alpha \beta }$ represents the slip hardening modulus matrix. When *α = β*, $h_{\alpha \beta }$ corresponds to the self-hardening modulus; when *α ≠ β*, $h_{\alpha \beta }$ signifies the latent hardening modulus. The self-hardening modulus is described by a power law function:

(6) $$h_{\alpha \beta }=h_{0}\sec h^{2}\left|h_{0}\gamma /(\tau_{s}-\tau_{0})\right|$$

where $h_{0}$ is the initial hardening modulus, $\tau_{0}$ is the initial critical shear stress, $\tau_{s}$ is the saturated flow stress, and γ is the cumulative shear strain of the open slip system:

(7) $$\gamma =\sum \int_{\alpha }\int_{0}^{t}\left|\gamma^{\left(\alpha \right)}\right|dt.$$

When the angle between *F***^p^** and *F***^e^** exists, the interactions of all slip systems are summed, as shown in the following equation:

(8) $$g^{\alpha }=\tau_{0}^{\alpha }+\mu b\sqrt{\sum_{\beta }G_{\alpha \beta }\rho^{\beta }}.$$

Dislocation density not only proliferates but also undergoes annihilation due to the interaction between slip systems. Therefore, the Kocks–Mecking (K-M) model is used to describe the dependence of dislocation density on strain rate [34]:

(9) $$\rho^{\alpha }=(k_{1}\sqrt{\sum_{\alpha =1}^{N}\rho }-k_{2}\rho^{\alpha })\cdot \left|\gamma^{\alpha }\right|.$$

The hardening relationship based on dislocation density is established through Equations (8) and (9) [35]:

(10) $$g^{\alpha }=\sum_{\beta =1}^{N}\frac{\mu bG_{\alpha \beta }}{2\sqrt{\sum_{\theta =1}^{N}G_{\alpha \theta }\rho^{\theta }}}(k_{1}\sqrt{\sum_{\theta =1}^{N}\rho^{\theta }}-k_{2}\rho^{\beta })\cdot \left|\gamma^{\beta }\right|$$

With this theoretical framework, the anisotropic plastic response of crystalline materials can be accurately predicted.

## 3. Experiments and Finite Element Modeling

### 3.1. Experiments

Based on the requirements of this study, nickel-based single-crystal superalloy NBSX (grade DD6) was selected as the experimental material, which was commercially supplied by Aero Engine Corporation of China, Beijing Institute of Aeronautical Materials (AECC BIAM). The general preparation steps are as follows.

Smelting: The master alloy of DD6 alloy is usually prepared by smelting in a vacuum induction furnace or vacuum arc furnace. By precisely controlling the proportion of raw materials and parameters such as temperature and time during the smelting process, the uniformity of the alloy composition is ensured, and the content of impurities and gases is reduced. Casting: The master alloy is made into casting blanks, and then single crystal castings are prepared by precision casting technology. Common methods include the spiral crystal selection method and the seed crystal method. The spiral crystal selection method is as follows: in a vacuum induction directional solidification furnace, the spiral channel is used to screen the grains, and only the grains growing along a specific direction (such as the (001) direction) can pass through the spiral channel, eventually forming a single crystal casting. The dendrite structure and element distribution of the alloy can be affected by controlling process parameters such as the pulling rate. Heat treatment: The castings are subjected to heat treatment, including solution treatment and aging treatment. The DD6 single crystal alloy test bar is placed in a vacuum directional solidification furnace for solution heat treatment first: it is heated to 1290 °C and held for 1 h, then heated to 1300 °C and held for 2 h, then further heated to 1315 °C and held for 4 h, followed by air cooling. Then, aging heat treatment is carried out: it is put into the furnace again, heated to 1100 °C and held for 4 h, then air-cooled, and then heated to 870 °C and held for 32 h, followed by air cooling. Heat treatment can regulate the morphology of the γ′ phase in the alloy, enabling it to achieve a better strengthening effect, thereby improving the high-temperature mechanical properties of the alloy. To meet the specific requirements of nanoindentation testing, specimens were first extracted from a [001]-oriented single-crystal ingot using precision wire-cutting technology, processing them into standard prismatic specimens measuring 10 mm (length) × 2 mm (width) × 6 mm (thickness). Subsequently, the specimens were embedded and cured using epoxy resin mounting powder in a vacuum mounting machine, ensuring structural stability during subsequent processing steps.

The specimen surface was processed using a graded precision grinding procedure: initial multi-stage mechanical grinding was performed on an automatic grinder employing 200#, 600#, 1000#, 1200#, 1500#, and 2000# silicon carbide (SiC) abrasive paper in sequence. Each grinding stage was rotated 90° from the previous orientation to eliminate unidirectional scratches. Subsequently, the specimen was transferred to an electrolytic polishing system where mirror finishing was achieved under constant voltage mode using a custom electrolyte, ultimately obtaining a test area with surface roughness Ra < 5 nm.

Nanoindentation testing was conducted on an Agilent Nanoindenter G200 system (Agilent Technologies, Santa Clara, CA, USA) equipped with a high-precision spherical diamond indenter (nominal radius R = 5.9 μm). Experiments employed load-controlled mode with a maximum load of 48 gf (converted to 470 mN). To investigate time-dependent deformation behavior, four distinct loading programs were specifically designed, featuring loading phase durations of 2000 s, 1000 s, 200 s, and 20 s, respectively. All programs synchronized a 10 s holding period and a 5 s linear unloading phase. Five repeat tests were performed for each loading parameter group to ensure data reliability, with adjacent indentations spaced at least 20 times the maximum indentation diameter apart to effectively prevent strain field interactions. Testing occurred within a constant temperature/humidity laboratory (23 ± 0.5 °C, relative humidity 45 ± 5%), with real-time thermal drift correction implemented via dynamic contact depth monitoring technology.

**Figure 1:**
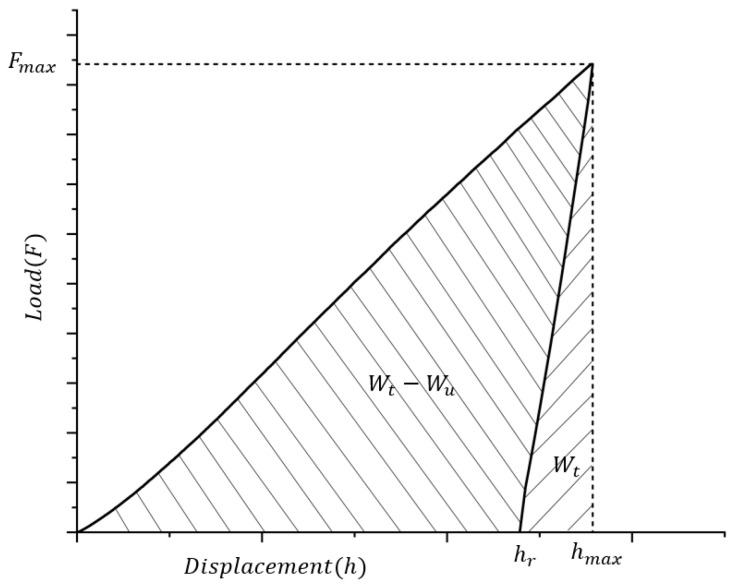
Nanoindentation loading–unloading curve and plastic work *W*_t_ − *W*_u_ and elastic energy *W*_u_.

Figure 1 shows the typical load–displacement (*F*-*h*) curve characteristics in nanoindentation tests with a spherical indenter. By integrating the area under the loading and unloading curves, the total mechanical energy *W*_t_ (corresponding to the integrated area of the curve in the loading stage) and the elastic recovery energy *W*_u_ (corresponding to the integrated area of the curve in the unloading stage) of the material during indentation can be quantified, respectively, and the difference between the two, *W*_t_ − *W*_u_, characterizes the energy dissipated by irreversible plastic deformation.

### 3.2. Finite Element Modeling

**Figure 2:**
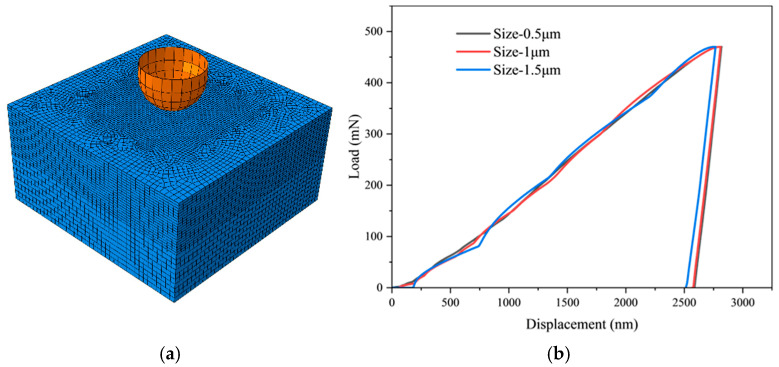
(**a**) CPFEM model. (**b**) Mesh sensitivity verification.

Considering the indentation depth, the size of the finite element model is 40 µm × 40 µm × 20 µm, each element is 0.5 μm × 0.5 μm × 0.5 μm in middle area with a size of 10 × 10 × 10 µm^3^, totaling 129,342 elements, the mesh is as shown in Figure 2a, and the cell type is C3D8 for the indenter modeled as a rigid shell with a radius of 5.9 µm. The mesh sensitivity verification under different mesh size conditions is conducted, as displayed in Figure 2b. When the mesh is 1.5 μm, a significant deviation is observed in the load–displacement curve in comparison to the 1 and 0.5 μm cases. Concurrently, fluctuations become discernible in the loading section when the mesh size is 1 and 1.5 μm. When the mesh is 0.5 μm, the curve loading section is smooth and aligns closely with the 1 μm mesh. Consequently, the grid size is designated as 0.5 μm. Simulation is divided into three stages: loading, holding, and unloading, and the loading speed is controlled by the loading time, consistent with the experiment and being 20 s, 200 s, 1000 s, 2000 s, respectively.

## 4. Results and Discussion

### 4.1. Experimental Results

Based on the theory of magnitude analysis [27], it is revealed that there is a significant linear correlation between the dimensionless parameters *h*_max_/R, *H*/E^*^ and the plastic energy share (*W*_t_ − *W*_u_)/*W*_t_, with the scaling factor dominated only by *h*_max_/R (R is the radius of curvature of the indenter, and *h*_max_ is the maximum depth of indentation). For a certain fixed *h*_max_/R, the relationship can be expressed as follows:

(11) $$\frac{\left(W_{t}-W_{u}\right)}{W_{t}}=B\frac{H}{E^{*}}+1$$

where *B* is obtained by applying a least-squares fit to the linear relationship between *H*/E* and (*W*_t_ − *W*_u_)/*W*_t_, while the value of B depends only on *h*_max_/R:

(12) $$B=-1.687{\left(\frac{h_{\max}}{R}\right)}^{-0.62}$$

Equations (11) and (12) lead to the following:

(13) $$\frac{H}{E^{*}}=0.5928{\left(\frac{h_{\max}}{R}\right)}^{0.62}\left(\frac{W_{u}}{W_{t}}\right)$$

Combine the well-known relationships between the reduced modulus E*, the initial unloading stiffness *S*, and the contact area *A*_c_:

(14) $$E^{*}=\frac{\sqrt{\pi }}{2}\frac{S}{\sqrt{A_{c}}}$$

Combining Equations (13) and (14) immediately establishes a multiparameter coupled energy–stiffness coupling equation, which realizes the analytical solution for the contact area *A*_c_, the reduced modulus E*, and the stiffness *H*. The coupled energy-stiffness equations are then solved for the contact area *A*_c_, the reduced modulus E*, and the stiffness *H*:

(15) $$A_{c}={\left[\frac{1.903F_{\max}}{{\left(\frac{h_{\max}}{R}\right)}^{0.62}\left(\frac{W_{u}}{W_{t}}\right)S}\right]}^{2}$$

(16) $$E^{*}=\frac{0.4657S^{2}{\left(\frac{h_{\max}}{R}\right)}^{0.62}\left(\frac{W_{u}}{W_{t}}\right)}{F_{\max}}$$

(17) $$H=0.276{\left(\frac{h_{\max}}{R}\right)}^{1.24}{\left(\frac{W_{u}}{W_{t}}\right)}^{2}\frac{S^{2}}{F_{\max}}$$

The core breakthrough of this methodology lies in its ability to simultaneously extract the material’s intrinsic elastoplastic parameters solely through mechanical response curves, without relying on conventional optical measurements of indentation morphology or empirical area functions. This theoretical framework provides a high-precision, low-damage solution for mechanical characterization of materials at the nanoscale.

**Figure 3:**
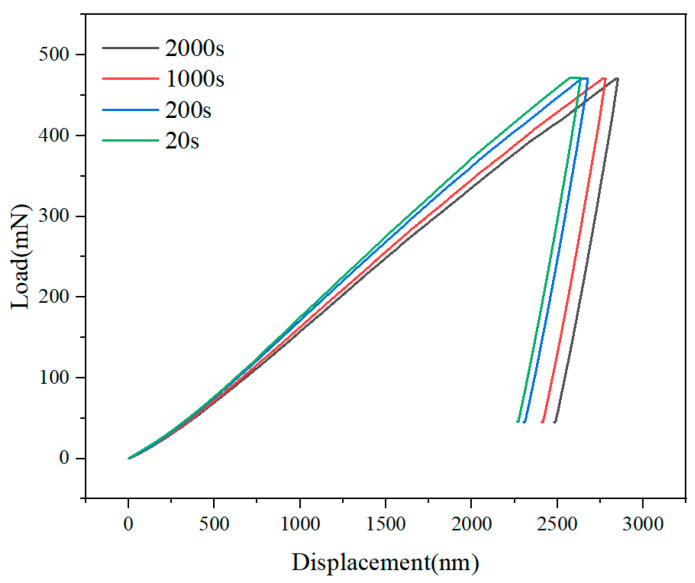
Load–displacement curves at various loading time.

Figure 3 presents the typical dynamic response curves of load (*F*) versus penetration depth (*h*) during nanoindentation. From comparative analysis of indentation curves under identical maximum load (*F*_max_ = 470 mN), the curves under varying loading rates (corresponding to loading times from 20 s to 2000 s) reveal significant time-dependent characteristics: as loading time increases from 20 s to 2000 s, the maximum penetration depth (*h*_max_) exhibits a systematic upward trend. This phenomenon directly demonstrates the sensitivity of DD6 superalloy to loading rate during nanoindentation, indicating a distinct positive strain-rate sensitivity (strain-rate sensitivity exponent *m* > 0). Further examination of curve morphology indicates that under prolonged loading conditions (e.g., 2000 s), the slope of the load-depth curve progressively decreases during the loading phase. Such time-dependent plastic flow enhances strain energy accumulation beneath the indenter. Constrained by the synergistic mechanisms of dislocation slip and diffusion creep, deformation energy cannot be dissipated efficiently through dynamic recovery. Consequently, this results in a larger residual indentation depth (*h*_r_) after unloading.

Through analyzing the load–displacement dynamic response characteristics at different load rates in Figure 3 and utilizing the energy method (Equations (15)–(17)) for multi-parameter simultaneous solving, key mechanical parameters of DD6 single-crystal superalloy were successfully extracted, including contact area *A*_c_ and hardness *H* (detailed in Table 2). Experimental data revealed a slight increase trend in material hardness *H* from 5.89 GPa to 6.09 GPa when the loading time decreased from 2000 s to 20 s (corresponding to a two-orders-of-magnitude increase in strain rate). This phenomenon implies the alloy’s strain-rate sensitivity at the nanoscale, the strengthening behavior is microscopically attributed to the transition in the dominant mechanism of dislocation slip under high strain rates: rapid loading restricts the activation time required for dislocation nucleation and multiplication. This forces the dislocation network to evolve within a smaller spatial scale, concurrently intensifying the interfacial stress concentration effect between the γ′ strengthening phase and the matrix. Collectively, these factors induce elevated resistance to plastic deformation. Previous studies have measured the hardness of this material to be in the range of 3.1–3.5 GPa using a conical indenter [13]. However, this paper uses a spherical indenter, which has a larger deformation area underneath the indenter and therefore yields a higher hardness value than conical indentation.

### 4.2. Crystal Plasticity Parameters

**Figure 4:**
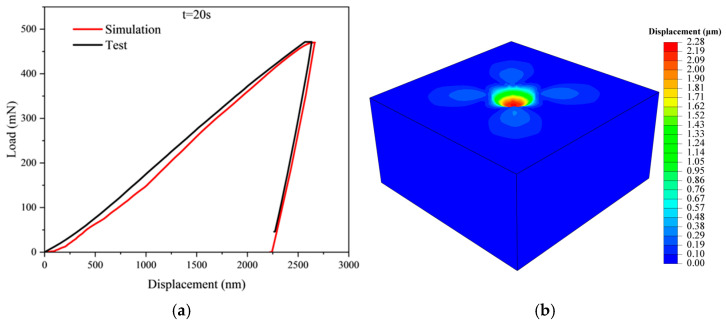
DD6 [001] orientation. (**a**) Comparison of experimental and CPFEM load–displacement curves, (**b**) stress cloud map.

Crystal plasticity finite element simulations employed a dislocation density-based constitutive model, where material hardening was determined by dislocation density multiplication and annihilation. The simulations were benchmarked against experimental load–displacement curves obtained at a 20 s loading time, with fitted and experimental results compared in Figure 4.

From Figure 4, it can be seen that the established crystal plasticity finite element model can better simulate the deformation process of the spherical indentation experiment, from which the response crystal plasticity parameters can be obtained, as shown in Table 3. By comparing the maximum indentation depth between simulation and experiment, when t = 20 s, the maximum D-value of the indentation depth is 38 nm with an error of 1.53%; when t = 200 s, the D-value is 161nm with an error of 5.76%; when t = 1000 s, the D-value is 149 nm with an error of 5.10%; and when t = 2000 s, the D-value is 187 nm with an error of 6.29%, respectively. Under the four loading conditions, the error rates are all less than 10%.

**Figure 5:**
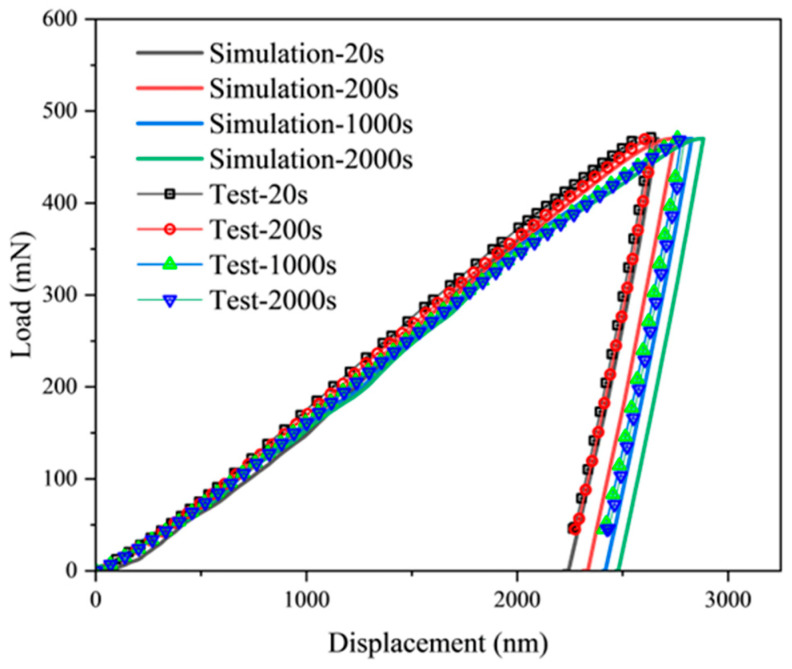
Load–displacement curves for different loading rates.

On the basis of the crystal parameters in Table 3, the load–displacement curves of DD6 [001] in the crystallographic direction under different loading time conditions can be further simulated and calculated, as shown in Figure 5. It can be seen that the results obtained from the simulation are basically in agreement with the experimental results.

### 4.3. Mechanical Characterization Under Different Loading Rates

**Figure 6:**
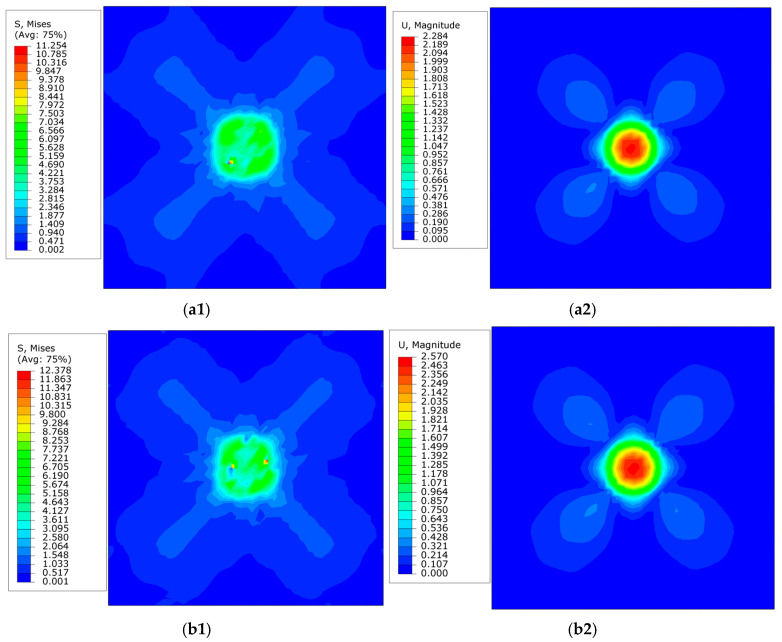
Stress and displacement cloud maps at unloading point. (**a**) Stress (**a1**) and displacement (**a2**) cloud maps for loading time of 20 s. (**b**) Stress (**b1**) and displacement (**b2**) cloud maps for loading time of 200 s.

For investigating the mechanical characteristics under different loading rate conditions, as shown in Figure 6, the simulation results were used to intercept the stress and displacement cloud diagrams of the unloading point at the loading time of 20 s and 200 s, respectively.

Spherical indentation simulations reveal distinct load–displacement curves under varying loading durations. Simulated results exhibit close alignment with experimental data at a 20 s loading time. As shown in Figure 6, the material displays significant anisotropic characteristics, uniformly manifesting a cross-shaped distribution pattern. Note that all images in Figure 6 represent post-unloading states: Figure 6a specifically visualizes residual stress and displacement contours. This anisotropic distribution arises from the crystallographic orientation of slip systems—their slip directions form specific angles with the model’s *x*- and *y*-axes. Consequently, the strengthening directions of these slip systems align diagonally, resulting in the observed X-shaped distribution of residual stress, displacement, and deformation.

**Figure 7:**
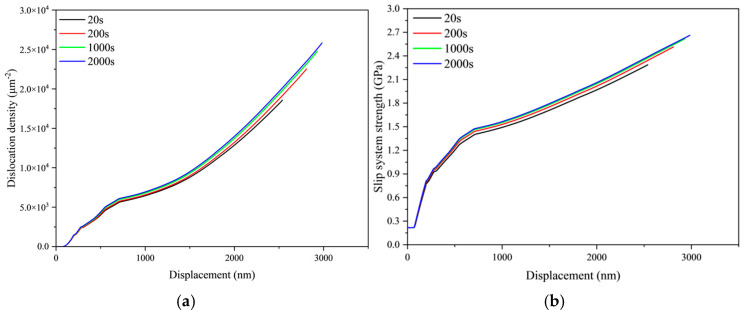
Evolution characteristics of crystal plasticity under different loading time conditions. (**a**) Dislocation density, (**b**) slip system strength.

Figure 7 illustrates the evolution of crystal dislocation density and slip system strength with indentation depth under varying loading times. The data indicate that the material enters its hardening stage at a displacement of 72 nm, where both dislocation density and slip system strength begin to increase, signifying the onset of plastic deformation. Material hardening results from the multiplication and annihilation of dislocations. Consequently, as deformation progresses, dislocation density and slip system strength exhibit gradual increases. These parameters also demonstrate distinct rate dependency: Under identical indentation depths, both metrics systematically rise with prolonged loading time—a trend fully consistent with the previously observed creep behavior.

### 4.4. Mechanical Characterization Under Different Crystal Orientations

**Figure 8:**
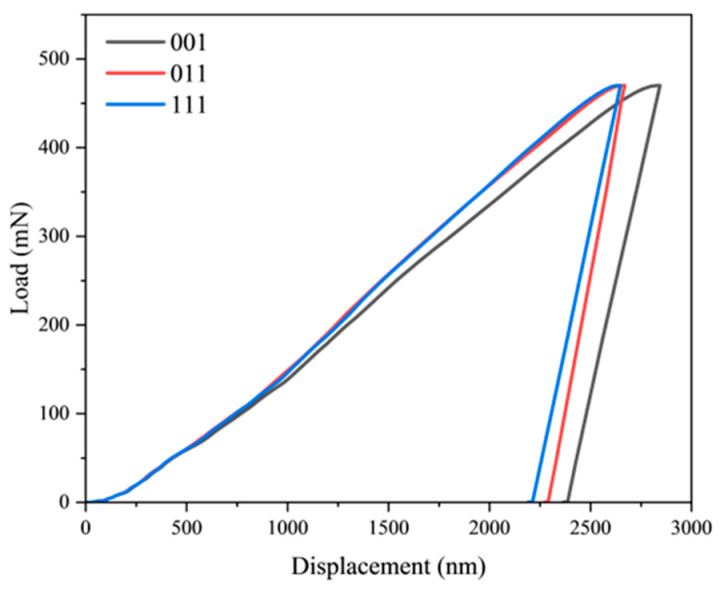
Load–displacement curves for different crystal orientations at a loading time of 200 s.

**Figure 9:**
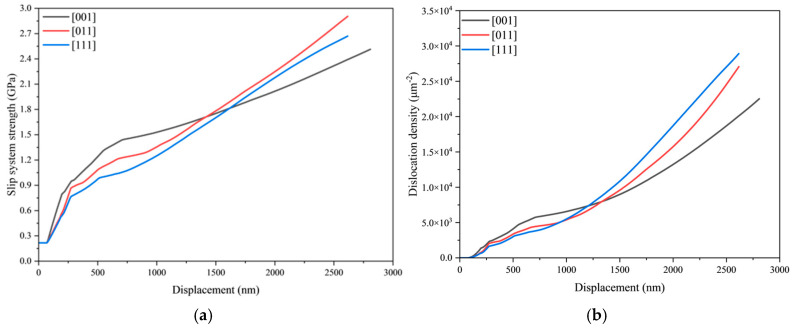
Relationship curves of slip system strength and dislocation density evolution with indentation depth for different crystal orientations. (**a**) Slip system strength; (**b**) Dislocation density.

To further investigate the indentation mechanical behavior under different crystallographic orientations, crystal plasticity finite element simulations were conducted for spherical indentation processes with crystal orientations of [011] and [111]. Figure 8 displays the load–displacement curves for the distinct crystallographic orientations at a loading time of 200 s. Figure 9 presents the evolution curves of slip system strength and dislocation density versus indentation depth for both orientations at the 200 s loading time.

Figure 9 reveals significant variations in the evolution patterns of slip system strength and dislocation density across different crystallographic orientations. Notably, the [011] and [111] orientations demonstrate remarkably similar mechanical responses, as evidenced by their nearly identical load–displacement curves in Figure 8. Comparative examination of Figure 8 and Figure 9 indicates that the [001] orientation exhibits both the lowest dislocation density and slip system strength, resulting in the largest indentation displacement among the three orientations. While the [011] and [111] orientations share similar trends in slip system strength, dislocation density, and load–displacement behavior, key differences emerge: the [011] orientation displays relatively higher slip system strength but lower dislocation density compared to [111]. These findings clearly demonstrate the substantial influence of material anisotropy on deformation behavior. In the same orientation, the material strength increases with increasing dislocation density, while comparisons between different orientations necessitate consideration of anisotropic effects.

**Figure 10:**
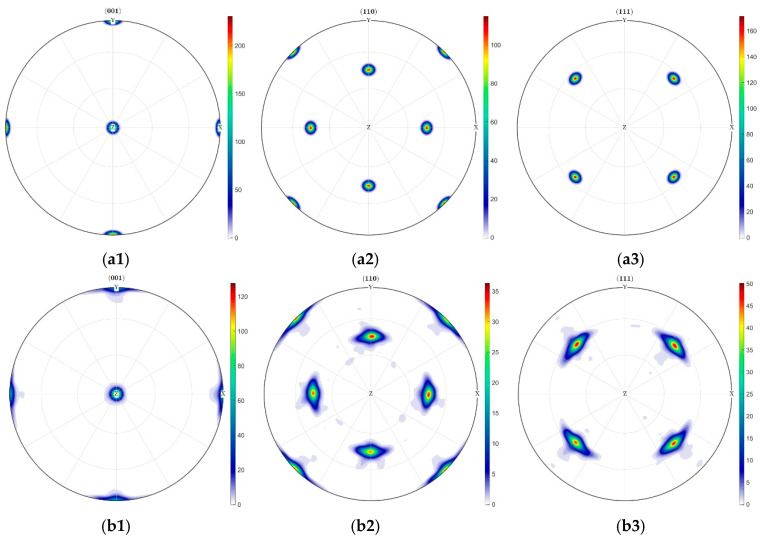
Texture densities of different crystal faces under [001] crystal orientation conditions. (**a**) Before indentation: (**a1**) Evolution pole figure of (001) crystal plane density distribution, (**a2**) Evolution pole figure of (110) crystal plane density distribution, (**a3**) Evolution pole figure of (111) crystal plane density distribution. (**b**) After indentation: (**b1**) Evolution pole figure of (001) crystal plane density distribution, (**b2**) Evolution pole figure of (110) crystal plane density distribution, (**b3**) Evolution pole figure of (111) crystal plane density distribution.

Furthermore, texture evolution was characterized through pole figures extracted from a central 12 μm × 12 μm indentation region in the CPFEM model, as presented in Figure 10. Texture evolution within the indentation zone of the [001]-oriented crystal demonstrates homogeneous characteristics with balanced crystal rotation. The maximum pole density of the (001) plane decreased from 200 to 120. Substantial diffuse scattering of other rotated crystal planes caused significant variations in their corresponding pole figures.

**Figure 11:**
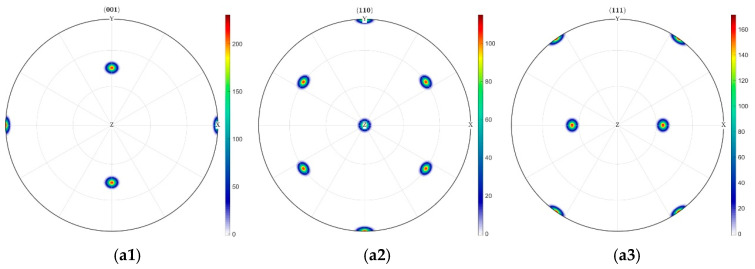

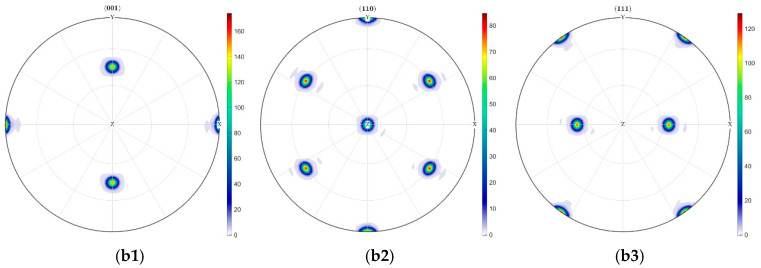
Texture densities of different crystal faces under [011] crystal orientation conditions. (**a**) Before indentation: (**a1**) Evolution pole figure of (001) crystal plane density distribution, (**a2**) Evolution pole figure of (110) crystal plane density distribution, (**a3**) Evolution pole figure of (111) crystal plane density distribution. (**b**) After indentation: (**b1**) Evolution pole figure of (001) crystal plane density distribution, (**b2**) Evolution pole figure of (110) crystal plane density distribution, (**b3**) Evolution pole figure of (111) crystal plane density distribution.

Figure 11 reveals directional texture evolution in the [011]-oriented indentation zone, where (001) planes undergo significant rotation toward the coordinate origin along the material’s *x*-axis, while other crystallographic planes exhibit preferential tilting toward the *z*-axis. Notably, the maximum density of (001) decreased from 200 to 160. The subsequent concentrated redistribution of poles following crystallographic rotation accounts for minimal variations in pole figures, with maximum density remaining essentially unchanged.

**Figure 12:**
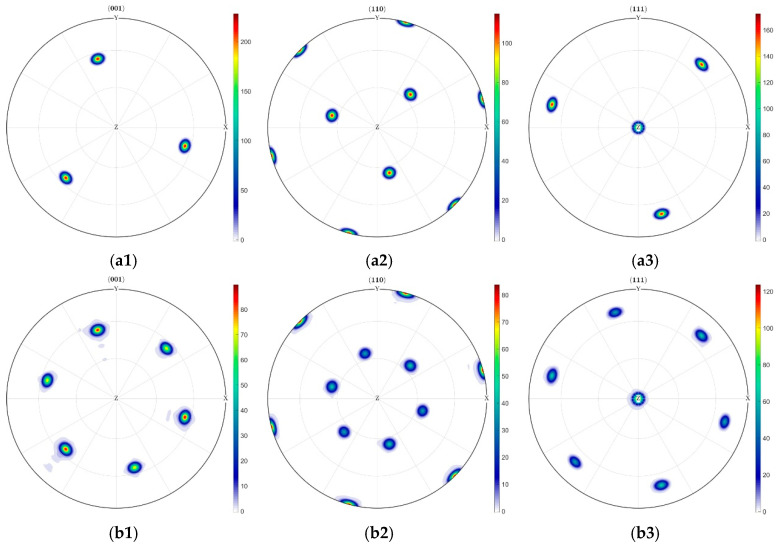
Texture densities of different crystal faces under [011] crystal orientation conditions. (**a**) Before indentation: (**a1**) Evolution pole figure of (001) crystal plane density distribution, (**a2**) Evolution pole figure of (110) crystal plane density distribution, (**a3**) Evolution pole figure of (111) crystal plane density distribution. (**b**) After indentation: (**b1**) Evolution pole figure of (001) crystal plane density distribution, (**b2**) Evolution pole figure of (110) crystal plane density distribution, (**b3**) Evolution pole figure of (111) crystal plane density distribution.

Figure 12 demonstrates more systematic texture evolution in the [111]-oriented indentation zone, exhibiting heightened rotational coherence and stronger directional preference. Except for the specimen-surface-parallel (111) planes, multiple crystallographic planes undergo coordinated rotation about the *z*-axis within a well-defined angular range of ±60°. Concurrently, the maximum density of (001) planes exhibit a sharp decline from 200 to 80. This rearrangement induces substantially enhanced pole figure variations. While maximum densities at equatorial and polar regions in (110) and (111) pole figures remain relatively stable, significant directional rotations occur in intermediate orientations. Crucially, these rotations demonstrate pronounced angular consistency and spatial regularity, indicative of deformation-mediated crystallographic reorientation.

Comparative analysis of texture evolution across three crystallographic orientations reveals significant anisotropy in material responses during indentation, manifested through distinct magnitudes of crystallographic rotation and varying degrees of rotational dispersion. This orientation dependence profoundly influences texture development, dislocation density, and slip system activity. Specifically, (111) pole figure distributions demonstrate a direct correlation between rotational intensity and material hardness: the [111] orientation exhibits maximal rotation corresponding to peak hardness, followed by intermediate rotation/hardness in [001], while the [011] orientation shows minimal rotation coinciding with the lowest hardness. The complex, multiaxial stress states inherent to nanoindentation trigger orientation-dependent activation of slip systems with variant slip magnitudes per system. Consequently, definitive orientation-governed patterns for slip system strength evolution and dislocation density distribution remain inadequately characterized due to this multifactorial stress superposition and resultant deformation heterogeneity.

### 4.5. Hardness and Contact Area of DD6 Under Different Loading Rates and Crystal Orientations

**Figure 13:**
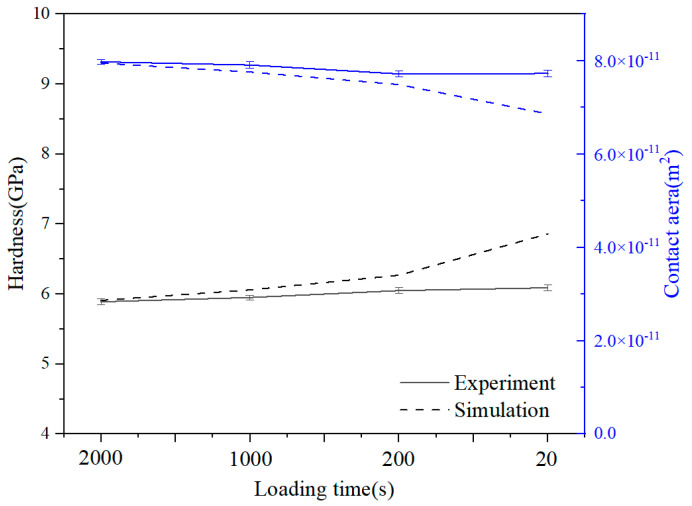
Comparison of indentation hardness and contact area obtained from experiments and CPFEM at different load rates.

Based on CPFEM simulations and nanoindentation theory, the indentation hardness and contact area under various crystallographic orientations and loading rates were calculated, as detailed in Table 4. Figure 13 demonstrates good agreement between the CPFEM predictions and experimental hardness measurements. The results reveal a distinct rate-dependence, with hardness progressively decreasing as loading time increases. At identical loading durations, material hardness varies significantly with crystallographic orientation: the [111] orientation exhibits maximum hardness, followed by [001], while [011] shows minimum values, clearly demonstrating material anisotropy. This anisotropic behavior is intrinsically linked to texture evolution within the indentation zone directly beneath the indenter tip. And due to the intrinsic viscoplastic characteristics of the adopted CPFE model for numerical simulation, the relatively larger strain-rate sensitivity of the simulations in comparison to the experimental data is shown.

## 5. Conclusions

This study investigates the nanoindentation behavior of nickel-based single-crystal superalloy DD6 under spherical indentation through combined experimental characterization and crystal plasticity finite element modeling (CPFEM). The dislocation density-incorporated CPFEM framework effectively simulates nonlinear indentation responses while quantitatively characterizing the evolutionary behavior of dislocation density and slip system resistance with penetration depth. Both the experimental and the simulation results suggest that the hardness of the crystal is sensitive to the loading rate. Significant mechanical heterogeneity exists across crystallographic orientations ([001], [011], [111]) in DD6. The [111] orientation exhibits the highest hardness, contrasting with the minimum value observed at [011]. This anisotropy correlates directly with the extent of lattice rotation during texture evolution. Specifically, the [111]-oriented crystal demonstrates highly localized and systematic lattice rotation, while the [001] orientation displays homogeneous texture development. These phenomena confirm that anisotropic deformation mechanisms are governed by differential slip system activation and crystallographic reorientation.

## Figures and Tables

**Table 1:** Chemical composition of DD6 alloy.

wt.% (Cr)	wt.% (Co)	wt.% (W)	wt.% (Mo)	wt.% (Al)
0.8~4.8	8.5~9.5	7.0~9.0	1.5~0.5	5.2~6.2
wt.% (Nb)	wt.% (Ta)	wt.% (Re)	wt.% (Hf)	wt.% (Ni)
0~1.2	6.0~8.5	1.6~0.4	0.05~0.1	Residuals

**Table 2:** Indentation hardness and contact area at different load rates.

Loading Time (s)	*H* (GPa)	*A*_c_ (m^2^)
2000	5.89 ± 0.042	7.98 × 10^−11^ ± 5 × 10^−13^
1000	5.95 ± 0.031	7.91 × 10^−11^ ± 7 × 10^−13^
200	6.05 ± 0.041	7.72 × 10^−11^ ± 6 × 10^−13^
20	6.09 ± 0.045	7.73 × 10^−11^ ± 7 × 10^−13^

**Table 3:** Crystal plasticity parameters of DD6.

Symbol	Description	Valve	Unit
$C_{11}$	Elastic constant	200	MPa
$C_{12}$	Elastic constant	138	MPa
$C_{44}$	Elastic constant	46	MPa
*n*	Rate sensitivity coefficient	25	-
${\dot{\gamma }}_{0}^{\left(\alpha \right)}$	Reference strain rate on α slip system	0.0005	s^−1^
ρ	Initial dislocation density	5000	mm^−2^
$\tau_{0}$	Critical shear stress	215	MPa
b	Burgers vector magnitude	2.53 × 10^−7^	mm
μ	Shear modulus	106.21	MPa
$k_{1}$	Multiplication coefficient of dislocation	305,000	mm^−1^
$k_{2}$	Annihilation coefficient of dislocation	2	-

**Table 4:** Hardness and contact area for different crystal orientations and loading rates.

Loading Time (s)	*H* (GPa)	*A*_c_ (m^2^)
2000	5.91	7.95 × 10^−11^
1000	6.06	7.76 × 10^−11^
200	6.27	7.49 × 10^−11^
20	6.86	6.86 × 10^−11^
200-(011)	6.07	7.11 × 10^−11^
200-(111)	6.57	7.15 × 10^−11^

## Data Availability

The original contributions presented in this study are included in the article. Further inquiries can be directed to the corresponding authors.
